# Inhibitory Potential of Quercetin Derivatives Isolated from the Aerial Parts of *Siegesbeckia pubescens* Makino against Bacterial Neuraminidase

**DOI:** 10.3390/molecules28145365

**Published:** 2023-07-12

**Authors:** Yun Gon Son, Ju Yeon Kim, Jae Yeon Park, Kwang Dong Kim, Ki Hun Park, Jeong Yoon Kim

**Affiliations:** 1 Department of Pharmaceutical Engineering, Institute of Agricultural and Life Science (IALS), Anti-Aging Bio Cell Factory Regional Leading Research Center (ABC-RLRC), Gyeongsang National University, Jinju 52725, Republic of Korea; thsdbsrhs112@naver.com (Y.G.S.); juy2755@naver.com (J.Y.K.); wpdus1883@naver.com (J.Y.P.); 2 Division of Applied Life Science (BK21 Four), Institute of Agricultural and Life Science (IALS), Anti-Aging Bio Cell Factory Regional Leading Research Center (ABC-RLRC), Gyeongsang National University, Jinju 52828, Republic of Korea; kdkim88@gnu.ac.kr (K.D.K.); khpark@gnu.ac.kr (K.H.P.)

**Keywords:** bacterial neuraminidase, *Siegesbeckia pubescens* Makino, quercetin derivatives, enzyme kinetics, binding affinity

## Abstract

This study aimed to isolate bacterial neuraminidase (BNA) inhibitory O-methylated quercetin derivatives from the aerial parts of *S. pubescens*. All the isolated compounds were identified as O-methylated quercetin (**1**–**4**), which were exhibited to be noncompetitive inhibitors against BNA, with IC_50_ ranging from 14.0 to 84.1 μM. The responsible compounds (**1**–**4**) showed a significant correlation between BNA inhibitory effects and the number of O-methyl groups on quercetin; mono (**1**, IC_50_ = 14.0 μM) > di (**2** and **3**, IC_50_ = 24.3 and 25.8 μM) > tri (**4**, IC_50_ = 84.1 μM). In addition, the binding affinities between BNA and inhibitors (**1**–**4**) were also examined by fluorescence quenching effect with the related constants (*K*_SV_, *K*_A_, and *n*). The most active inhibitor **1** possessed a *K*_SV_ with 0.0252 × 10^5^ L mol^−1^. Furthermore, the relative distribution of BNA inhibitory O-methylated quercetins (**1**–**4**) in *S. pubescens* extract was evaluated using LC-Q-TOF/MS analysis.

## 1. Introduction

Neuraminidase (sialidase, EC 3.2.1.18), belonging to the hydrolases class, is mainly present in bacteria and viruses [1,2]. They are responsible for the cleavage of glycol-conjugated proteins on the host cell membrane to release sialic acid [3,4,5]. In particular, bacterial neuraminidase (BNA) contributes to bacterial cell-to-cell signaling [6], which leads to the generation of self-extracellular polymeric substances (EPSs) that cause bacterium to adhere to one another, which is known as biofilms [7,8]. In addition, neuraminidase is also secreted by bacterial pathogens for the colonization of biofilms [9], which causes differences in the immune defense system by being present in bacterially infected sites of the human body [10]. Depending on the infecting pathogen, BNA can cause various inflammatory diseases, including pneumonia, enteritis, and sepsis [11]. Although antibiotics provide an effective treatment for pathogenic infections, biofilms within pathogens provide protection from the antibacterial effects, enabling their survival [12,13,14]. Therefore, preventing the formation of biofilm through the inhibition of BNA activity is essential for the successful treatment of infectious, chronic inflammatory diseases [15]. Many recent studies have reported on the development of bacterial neuraminidase inhibitors from various natural sources, such as chromenone derivatives from *F. philippinensis* [16], ugonins from *H. zeylanica* [17], anthraquinone from *P. cuspidatum* [18], rotenoids from *A. fruticose* [19], etc.

*Siegesbeckia pubescens* is an annual plant native to East Asia, mainly Korea, Japan, and China [20]. The plant typically grows naturally in mountain and field areas [21], where it is collected during mature flowering for use in traditional medicine [22]. The dried plants of the aerial parts have been used as a remedy for various inflammations related to bones, joints, muscles, and rheumatoid pain [23]. From many previous reports, the therapeutic effects of *S. pubescens* are known to be associated with the well-known abundance of secondary metabolites, such as diterpenes, sesquiterpenes, and quercetin derivatives [24]. There is evidence of anti-inflammation effects from previous reports, which show that terpenes have the potential to inhibit Pam_3_CSK_4_-induced inflammation [25], reduce oxidation stress in vivo [26], inhibit elastase release [27], and attenuate postoperative inflammation. Quercetin derivatives also play a role in anti-inflammatory effects by inhibiting the production of pro-inflammatory cytokines and mediators, such as nitric oxide and prostaglandin E2 [20,28]. In particular, O-methylated quercetins in *S. pubescens* have not been reported to have BNA inhibitory capacities.

This study aimed to explore the inhibitory effects of O-methylated quercetins from the aerial parts of *S. pubescens* against BNA to evaluate its anti-inflammatory ability. Four O-methylated quercetins (**1**–**4**) responsible for BNA inhibition were isolated and identified using spectroscopic data. The kinetic studies of inhibitors were characterized by double-reciprocal plots against BNA. Binding affinities between BNA and isolated compounds were investigated by fluorescence quenching. Moreover, the natural abundance of BNA inhibitors (**1**–**4**) from the aerial parts of *S. pubescens* were analyzed using LC-Q-TOF/MS.

## 2. Results and Discussion

### 2.1. Structural Identification of O-Methylated Quercetins from S. pubescens

**Figure 1:**
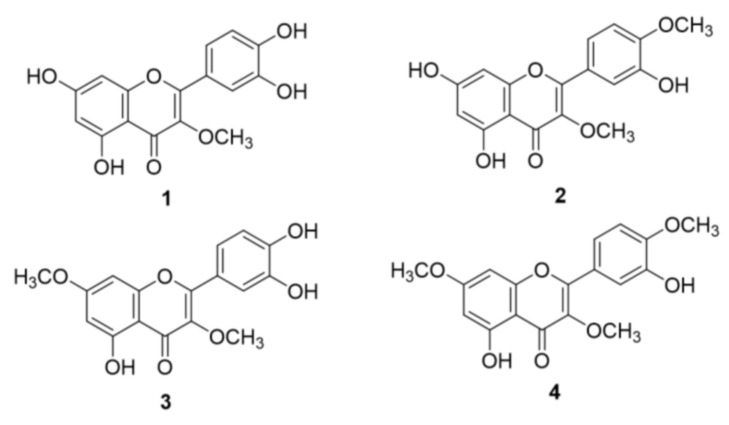
The isolated O-methylated quercetins (**1**–**4**) from the aerial parts of *S. pubescens*.

In the course of searching for a lead structure for bacterial neuraminidase (BNA), quercetin derivatives were isolated from the methanol extract of the aerial part of *S. pubescens*. From the crude extract, four quercetin derivatives (**1**–**4**) were isolated and purified through repetitive normal- and reverse-phase silica gel open columns and Sephadex LH-20. As shown in Figure 1, the chemical structures were completely identified as 3-*O*-methyl quercetin (**1**), 3,4′-*O*-dimethyl quercetin (**2**), 3,7-*O*-dimethyl quercetin (**3**), and 3,7,4′-*O*-trimethyl quercetin (**4**). Compound **1** was obtained as a yellow powder, with the molecular formula C_16_H_12_O_7_ determined by HRESIMS (*m*/*z* 317.0642 [M + H]⁺, calculated as 317.0661). The general structure of quercetin was confirmed by proton NMR. The resorcinol moieties on the A-ring were represented by H-6 (δ_H_ 6.10, d, *J* = 1.6 Hz) and H-8 (δ_H_ 6.27, d, 1.6 Hz). The catechol motifs on the B-ring were deduced from H-2′ (δ_H_ 7.53, d, *J* = 1.6 Hz), H-5′ (δ_H_ 6.80, d, *J* = 8.5 Hz), and H-6′ (δ_H_ 7.42, dd, *J* = 8.5, 2.0 Hz). One O-methyl group was verified by a singlet peak at δ_H_ 3.67 (3H, s, 3-OCH_3_). The position of the O-methyl (OCH_3_) group was deduced from the HMBC correlation between C-3 (δ_C_ 138.2) and OCH_3_. Thus, compound **1** was identified as 3-*O*-methyl quercetin.

### 2.2. Inhibitory Effects of Quercetin Derivatives against Bacterial Neuraminidase

**Figure 2:**
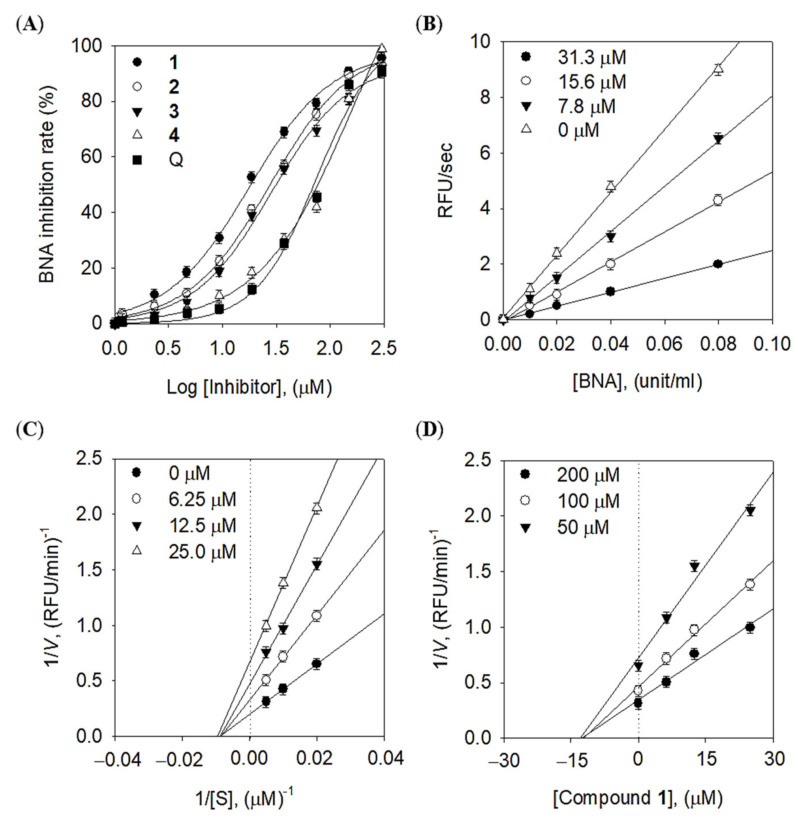
Enzyme kinetics of quercetin derivatives against BNA. (**A**) Dose-dependent inhibitory courses of BNA inhibitors (**1**–**4**) and positive control (quercetin), (**B**) reversibility, (**C**) lineweaver–Burk plot, and (**D**) Dixon plot of compound **1**.

Bacterial neuraminidase plays an important role through the production of sialic acid from host cells. Thus, inhibition of BNA was related to bacterial infectious inflammation. All of the isolated quercetin derivatives (**1**–**4**) exhibited dose-dependent inhibitory effects against BNA, with IC_50_ values of 14.0~84.1 μM (Figure 2A). The number of O-methyl groups showed a similar trend, with the following inhibitory effects (IC_50_): 14.0 μM 3-*O*-methyl quercetin (**1**) >25.8 μM 3,4′-*O*-dimethyl quercetin (**2**), and 24.3 μM 3,7-*O*-dimethyl quercetin (**3**) >84.1 μM 3,7,4′-*O*-trimethyl quercetin (**4**). The most active BNA inhibitor was compound **1**, which contained an O-methyl group. However, no differences in inhibitory effects were observed between compounds **2** and **3** with O-dimethyl moieties. Among them, the lowest level of activity was detected in O-trimethyl quercetin **4**. A higher level of inhibitory activity against BNA was detected for 3-*O*-methyl quercetin, compared with quercetin (IC_50_ = 26.1 µM) as a positive control, which had the mother skeleton of compound **1** (Table 1).

In addition, the inhibition modes and related constants of inhibitors were verified by double-reciprocal plots, including Lineweaver–Burk and Dixon plots. All of the isolated (O-methylated) quercetins (**1**–**4**) were confirmed as noncompetitive inhibitors, which exert inhibitory activities by binding to the enzyme–substrate complex. In the Lineweaver–Burk plot, the *x*-axis is the reciprocal of the substrate concentration derived from the *K*_m_ value, and the *y*-axis is the reciprocal of the value for maximum velocity. As shown in Figure 2C, the constant *K*_m_ value and the decreasing *V*_max_, indicating a noncompetitive mode, were observed for inhibitor 1, which was the most active. In addition, the enzyme inhibition constant (*K*_i_) was obtained by the determination of substrate concentrations between inhibitor concentrations and *V*_max_ values in the Dixon plot. Thus, it was confirmed that the *K*_i_ value of inhibitor **1** was 13.8 μM (Figure 2D). Other inhibitors (**2**–**4**) had *K*_i_ values of 24.7, 22.4, and 79.5 μM, respectively. In comparison with O-methyl substituents on the quercetin, the BNA inhibitory activities increased in the order of mono > quercetin, and di- > tri-. Four O-methylated quercetins (**1**–**4**) exhibited noncompetitive inhibition modes, where they were bound to an allosteric site on an enzyme complex, with the substrate confirmed by Lineweaver–Burk plots. Moreover, *K*_i_ values derived from the Dixon plot showed they were similar to IC_50_, reverifying them as noncompetitive inhibitors. Overall, 3-*O*-methyl quercetin (**1**) was the lead BNA inhibitor and was a more active metabolite than none, di-, or tri-type methylated quercetins.

### 2.3. Binding Affinity between Inhibitors and Enzyme

**Figure 3:**
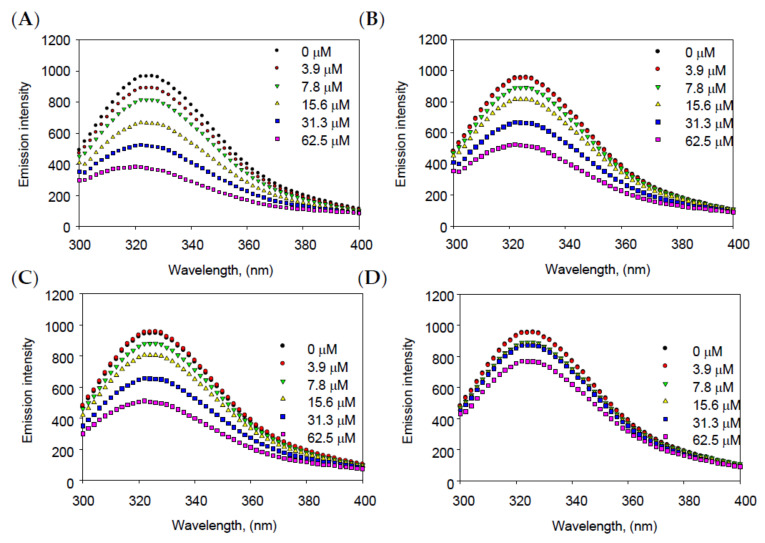
The emission spectra of binding affinity between BNA and inhibitors (**A**) **1**, (**B**) **2**, (**C**) **3**, and (**D**) **4**.

As a protein, bacterial neuraminidase contains specific fluorescent amino acid residues, including tyrosine, tryptophan, and phenylalanine. Therefore, the binding affinity between BNA and the inhibitors was assessed by confirming, at the emission wavelength, that the fluorescence of the enzyme was eliminated as inhibitors were bound to BNA [29]. In addition, various parameters of binding affinity, including *K*_SV_, *K*_A_, and *n*, were derived using Stern–Volmer, as shown in Equations (2) and (3). The fluorescence quenching effects were closely associated with the inhibitory effects (IC_50_) of inhibitors **1**–**4**. Compound **1**, the most active inhibitor, caused a greater reduction in fluorescence intensities, compared with di- and tri-*O*-methyl quercetins (**2**–**4**) at the same concentrations of 0 to 62.5 μM (Figure 3).

As shown in Table 2, using the Stern–Volmer constant (*K*_SV_), the tendencies were observed as inversely related to inhibitory effects against BNA (IC_50_), whereas the binding affinity constant (*K*_A_) showed a proportional correlation with IC_50_ values: **1** (*K*_SV_ = 0.0252 × 10^5^ L mol^−1^ and *K*_A_ = 0.05105 × 10^6^ L mol^−1^), **2** (*K*_SV_ = 0.0144 × 10^5^ L mol^−1^ and *K*_A_ = 0.03994 × 10^6^ L mol^−1^), **3** (*K*_SV_ = 0.0153 × 10^5^ L mol^−1^ and *K*_A_ = 0.03818 × 10^6^ L mol^−1^), and **4** (*K*_SV_ = 0.0036 × 10^5^ L mol^−1^ and *K*_A_ = 0.00021 × 10^6^ L mol^−1^). Additionally, all the Stern–Volmer constants (*K*_SV_) as indicators of binding affinities strongly correlated (R^2^ > 0.99) with the IC_50_ of BNA inhibitors. In addition, the number of binding sites (*n*) also showed the highest value at 3-*O*-methyl quercetin (**1**), compared with the others (**2**–**4**). Therefore, the results provided substantial evidence that the inhibitors could successfully bind to BNA through enzyme fluorescence quenching.

### 2.4. LC-Q-TOF/MS Analysis of S. pubescens Extract

**Figure 4:**
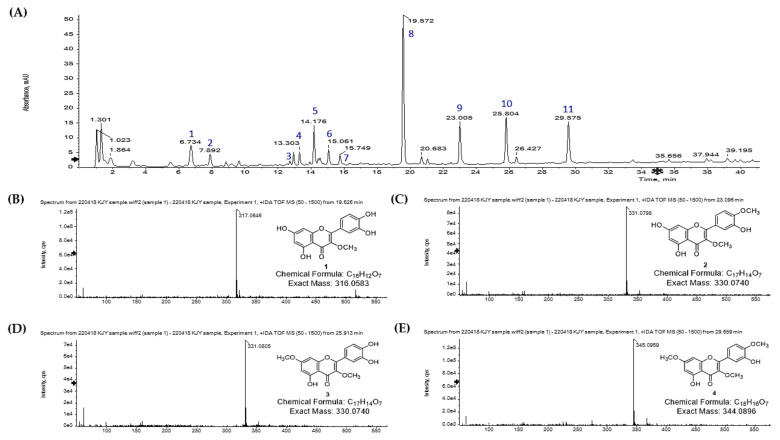
Analysis of *S. pubescens* extract using LC-Q-TOF/MS. (**A**) DAD chromatogram of the extract. Individual mass gram of (**B**) peak 8, (**C**) peak 9, (**D**) peak 10, and (**E**) peak 11.

*S. pubescens* extract contains both secondary metabolites and quercetin derivatives [30]. Thus, the distribution of quercetin derivatives from *S. pubescens* was confirmed by LC-Q-TOF/MS analysis. As shown in Figure 4, prominent peaks were observed at peaks 8 (*t*_R_ = 19.6 min), 9 (*t*_R_ = 23.0 min), 10 (*t*_R_ = 25.8 min), and 11 (*t*_R_ = 29.6 min), assigned as quercetin derivatives **1**–**4**, respectively. The most abundant peak 8 (*t*_R_ = 19.6 min) from the extract was identified as 3-*O*-methyl quercetin (**1**) by confirming the reliable error value between the observed ion at *m*/*z* 317.0642 and the calculated ion at *m*/*z* 317.0661. For peaks 9 (*t*_R_ = 23.0 min) and 10 (*t*_R_ = 25.8 min), similar molecular ions were observed at *m*/*z* 331.0798 and 331.0805, which corresponded with the calculated ion at [M + H]^+^ *m*/*z* 331.0818 of O-dimethyl quercetin (C_25_H_24_O_12_). By comparing the retention times of the isolated authentic compounds, peaks 9 and 10 were finally identified as 3,4′-*O*-dimethyl quercetin (**2**) and 3,7-*O*-dimethyl quercetin (**3**), respectively. Peak 10 was identified as 3,7,4′-*O*-trimethyl quercetin (**4**), which obtained the [M + H]^+^ *m*/*z* 345.0961, with an error value of −3.77 ppm in comparison with the calculated ion [M + H]^+^ *m*/*z* 345.0974. Other detected peaks, except for 8–11, were identified as glycosylated quercetins and phenolic acids by confirming the reasonable error values between detected and calculated ions (Table 3 and Supplementary Materials). The metabolites from *S. pubescens* identified using LC-Q-TOF/MS were as follows: peak 1, chlorogenic acid; peak 2, caffeic acid; peak 3, rutin; peak 4, isoquercitrin; peak 5, 3,5-*O*-dicaffeoylquinic acid; peak 6, 1,5-*O*-dicaffeoylquinic acid; peak 7, 3,4,5-tricaffeoylquinic acid; peak 8, 3-*O*-methyl quercetin; peak 9, 3,4′-*O*-dimethyl quercetin; peak 10, 3,7-*O*-dimethyl quercetin; and peak 11, 3,7,4′-*O*-trimethyl quercetin. As a result, BNA inhibitory compounds (**1**–**4**) were predominantly metabolites from the aerial part of *S. pubescens*. In particular, the most active compound **1** was the most abundant metabolite in the plant. Therefore, the qualitative data for quercetin derivatives can be valuable when developing *S. pubescence* as a promising anti-inflammatory nutraceutical.

## 3. Materials and Methods

### 3.1. Plant Materials and Chemicals

Collection of the aerial part of *S. pubescens* was conducted at a local mountain in Chungcheongbuk-do, Republic of Korea in March 2022. At that time, *S. pubescens* was in the yellow flowers blooming growth stage. The collected plant was dried under dark conditions at room temperature for the isolation of the metabolites. Quercetin, methanol-*d*_4_, chloroform-*d*, neuraminidase from *Clostridium perfringens*, 2′-(4-methylumbelliferyl)-α-d-*N*-acetylneuraminic acid sodium salt hydrate, and dimethyl sulfoxide were purchased from Sigma Aldrich (St. Louis, MO, USA). Organic solvents, including hexane, chloroform, ethyl acetate, acetone, and methanol used for the separation, isolation, and purification of metabolites were purchased from Daejung Chemical and Metals (Siheung-si, Gyeonggi-do, Republic of Korea). Analytical-grade acetonitrile and water were purchased from Honeywell (Charlotte, NC, USA).

### 3.2. Instruments

Proton (^1^H), carbon (^13^C), correlation spectroscopy (^1^H-^1^H COSY), heteronuclear single quantum coherence (^1^H-^13^C HSQC), heteronuclear multiple-bond correlation (^1^H-^13^C HMBC), and distortionless enhancement by polarization transfer spectra (DEPT 90 and 135) were recorded using AM 500 NMR (Bruker, Karlsruhe, Germany). Determination of the enzymatic inhibitory activity was performed on an iD3 spectrophotometer, which supported the automatic kinetic and fluorescence modes in the software Softmax 5.4.1 (Molecular devices, Sunnyvale, CA, USA). The chemical composition and natural abundance of O-methylated quercetins from *S*. *pubescens* were confirmed using X500R TOF/MS (SCIEX, Framingham, MA, USA).

### 3.3. Extraction, Separation, and Isolation of Quercetin Derivatives from S. pubescens

As the target of the isolation study, O-methylated quercetin was synthesized through the methylation of quercetin from the shikimate pathway in the plant. The several specialized enzymes, such as O-methyltransferase, were involved in the methylation by affecting the distributions of the plants. The dried aerial parts of *S. pubescens* (1 kg) with methanol (2 L × 3) were extracted at room temperature for two weeks. To remove chlorophyll from the plant, the crude extract (9.5 g) was injected into a Diaion HP-20 (Supelco, St. Louis, MO, USA) open column to elute it with methanol and acetone. Only the methanol layer was evaporated and dissolved in water, then separated with *n*-hexane, chloroform, ethyl acetate, and butanol, according to polarity. Among them, the ethyl acetate layer (3.8 g) was loaded on a silica gel (40~63 μm, Millipore, Burlington, MA, USA) open column (25 × 500 mm, 100 g) spilling chloroform and methanol solution (500:1 → 1:1) to afford nine fractions (Fr. A~Fr. I). Fr. D (210 mg) was subjected to Sephadex LH-20 with 80% methanol to afford compound **1** (28 mg). Fr. E (638 mg) was chromatographed with a reversed silica gel open column (Triart-prep C18, S-10, 12 nm, YMC, Kyoto, Japan), eluting it with a 75% acetonitrile isocratic condition to afford compounds **2** (37 mg) and **3** (18 mg), respectively. Fr. F (129 mg) was purified using Sephadex LH-20 to afford compound **4** (22 mg). Four isolated quercetin derivatives were identified as 3-*O*-methyl quercetin (**1**), 3,4′-*O*-methyl quercetin (**2**), 3,7-*O*-methyl quercetin (**3**), and 3,7,4′-*O*-trimethyl quercetin (**4**), respectively. Spectroscopic data regarding the compounds (**1**–**4**) are shown below.

#### 3.3.1. 3-*O*-Methyl Quercetin (**1**)

Yellow powder. HRESIMS [M + H]⁺ 317.0642 (calculated for C_16_H_12_O_7_, 317.0661). ^1^H NMR (500 MHz, MeOD): *δ*_H_ 3.67 (3H, s, 3-OCH_3_), 6.10 (1H, d, *J* = 1.6 Hz, H-6), 6.27 (1H, d, *J* = 1.6 Hz, H-8), 7.53 (1H, d, *J* = 1.6 Hz, H-2′), 6.80 (1H, d, *J* = 8.5 Hz, H-5′), and 7.42 (1H, dd, *J* = 8.5, 2.0 Hz, H-6′).

#### 3.3.2. 3,4′-*O*-Dimethyl Quercetin (**2**)

Yellow amorphous powder. HRESIMS [M + H]⁺ 331.0798 (calculated for C_17_H_14_O_7_ 331.0818). ^1^H NMR (300 MHz, MeOD): *δ*_H_ 3.81 (3H, s, 3-OCH_3_), 3.96 (3H, s, 4′-OCH_3_), 6.21 (1H, d, *J* = 2.1 Hz, H-6), 6.42 (1H, d, *J* = 2.1 Hz, H-8), 6.96 (1H, d, *J* = 8.5 Hz, H-5′), 7.64 (1H, dd, *J* = 2.0, 8.5 Hz, H-6′), and 7.73 (1H, d, *J* = 2.0, H-2′).

#### 3.3.3. 3,7-*O*-Dimethyl Quercetin (**3**)

Yellow amorphous powder. HRESIMS [M + H]⁺ 331.0805 (calculated for C_17_H_14_O_7_ 331.0818). ^1^H NMR (300 MHz, MeOD): *δ*_H_ 3.69 (3H, s, 3-OCH_3_), 3.77 (3H, s, 7-OCH_3_), 6.21 (1H, d, *J* = 2.1 Hz, H-6), 6.47 (1H, d, *J* = 2.1 Hz, H-8), 6.80 (1H, d, *J* = 8.5 Hz, H-5′), 7.44 (1H, dd, *J* = 1.9, 8.5 Hz, H-6′), and 7.54 (1H, d, J = 1.9 Hz, H-2′).

#### 3.3.4. 3,7,4′-*O*-Trimethyl Quercetin (**4**)

Yellow amorphous powder. HRESIMS [M + H]⁺ 345.0961 (calculated for C_18_H_16_O_7_ 345.0974). ^1^H NMR (300 MHz, CDCl_3_): *δ*_H_ 3.78 (3H, s, 3-OCH_3_), 3.80 (3H, s, 7-OCH_3_), 3.91 (3H, s, 4′-OCH_3_), 6.28 (1H, d, *J* = 2.2 Hz, H-6), 6.37 (1H, d, *J* = 2.2 Hz, H-8), 6.97 (1H, d, *J* = 8.4 Hz, H-5′), 7.59 (1H, dd, *J* = 1.8, 8.4 Hz, H-6′), and 7.63 (1H, d, *J* = 1.8 Hz, H-2′).

### 3.4. Bacterial Neuraminidase Inhibition Assay and Kinetics

Measurement of the inhibition of bacterial neuraminidase (BNA) activity was performed according to previous reports [29]. The BNA assay was performed using fluorescence (FS) of excitation and emission at 365 nm and 450 nm, respectively. At the fixed wavelength, the production of umbelliferon from sodium 2-(4-methylumbelliferyl)-*N*-acetylneuraminate as a florescent substrate was monitored for the determination of BNA activities. Reaction solutions consisted of 50 mM sodium acetate buffer, different concentrations of the isolated compounds or positive control (quercetin), 100 μM substrate, and 0.2 unit/mL neuraminidase from *Clostridium perfringens*. The mixture was placed in a 96-well black plate, and measurements of the fluorescence intensities per 30 sec until 30 min was performed using the equipped option in the spectrophotometer. The results were visualized and obtained using the connected software known as SoftMax Pro 5.4.1. BNA inhibitory potentials of the isolated quercetin derivatives (**1**–**4**) were calculated from the equation shown below (1).

BNA inhibition rate (%) = [(FS intensity of control − FS intensity of inhibitor)/FS in tensity of control] × 100(1)

Double-reciprocal plots were used to elucidate the results of kinetic studies of BNA inhibitory activities. Inhibitory modes were determined using the Lineweaver–Burk plot. The inhibition constant was derived from the origin of the Dixon plot. The reciprocal plots were displayed using the relationship between the Michaelis–Menten constant (*K*_m_) and the maximal velocity (*V*_max_), according to different concentrations of inhibitors, based on the half-maximal inhibitory concentration (IC_50_) value. The progressive curves were visualized using Sigma Plot ver. 10.0.

### 3.5. Fluorescence Quenching Experiments

BNA contains specific amino acid residues, including tryptophan, phenylalanine, and tyrosine, possibly as fluorescence quenching. In the interaction between the enzyme and the inhibitor, three amino acid residues showed reduced fluorescence intensities near 360 nm of emission wavelength, as indicated by the 290 nm of excitation wavelength. For the FQ analysis, 10 μL of 0.2 unit/mL neuraminidase from *Clostridium perfringens* and 10 μL of various concentrations of inhibitors containing individual IC_50_ ranges were mixed in 180 μL of sodium acetate buffer without substrate. Based on the results, the related parameters were calculated as follows, using Equations (2) and (3).

*F*_0_ − *F* = 1 + *K*_SV_ [Q] (2)

Log [(*F*_0_ − *F*)/*F*] = log *K*_A_ + n log [Q]f (3)

where *F*_0_ and *F* are fluorescence emission intensities in the absence and presence of quercetin derivatives, respectively; [Q] is the concentration of quenchers with the same means as the inhibitors; *K*_SV_ is the Stern–Volmer quenching constant; *K*_A_ is the binding constant between the enzyme and the quenchers; and n is the binding number between the enzyme binding site and the inhibitors.

### 3.6. LC-Q-TOF/MS Analysis

Shimadzu NEXERA (Shimadzu, Kyoto, Japan) was used as the equipment for LC analysis, and a Poroshell 120 EC-C18 column (2.1 mm × 100 mm, 2.7 µm, Agilent, Santa Clara, CA, USA) was used as the column for analysis. Purified water (A) containing 0.1% acetic acid and acetonitrile (B) containing 0.1% acetic acid were used as the mobile phase, with a flow rate of 1 mL/min. The solvent condition utilized a gradient solvent system that increased the ratio of the mobile phase composition B from 0 to 100% for 40 min. SCIEX X500R Q-TOF equipment was used for Q-TOF/MS, and MS conditions were set to 5.5 kV for capillary voltage and 450 °C for temperature in positive ionization mode. MS conditions were set to a collision energy of 10 V and a desolvation gas flow of 800 L/h at a temperature of 400 °C.

### 3.7. Statistical Analysis

Measurements of inhibitory activities against BNA, enzyme kinetics, and binding affinities were performed in triplicate. The mean, deviations, and *p*-values (<0.05) obtained in the results were expressed using SigmaPlot ver. 10.0 (Systate Software Inc., Chicago, IL, USA).

## 4. Conclusions

In this study, BNA inhibitory O-methylated quercetins were isolated from the aerial parts of *S. pubescens*. The chemical structures of isolated compounds were identified as 3-*O*-methyl quercetin (**1**), 3,4′-*O*-dimethyl quercetin (**2**), 3,7-*O*-dimethyl quercetin (**3**), and 3,7,4′-*O*-trimethyl quercetin (**4**) by fully spectroscopic data. BNA inhibitory effects showed a close relationship with the number of methyl groups on quercetin: **1** (IC_50_ = 14.0 μM, mono) > **2** (IC_50_ = 25.8 μM, di), and **3** (IC_50_ = 24.3 μM, di) > **4** (IC_50_ = 84.1 μM, tri). All of the compounds (**1**–**4**) were identified as noncompetitive inhibitors, which bind with the enzyme and substrate complex. In addition, inhibitors (**1**–**4**) that showed that binding affinities (*K*_SV_) with BNA were closely associated with inhibitory effects against BNA (IC_50_). Finally, the natural abundance of quercetin derivatives from *S. pubescens* extract was confirmed by the result showing that the most active BNA inhibitor (3-*O*-methyl quercetin, **1**) contained the highest contents of BPC using LC-Q-TOF/MS. This was the first report where O-methylated quercetins were responsible for BNA inhibition in the appropriate skeleton. The findings of this study suggest that the active metabolites from the plant have the potential to be used as anti-inflammatory lead compounds to associate with pathogenesis from bacterial infection. Further studies are necessary to elucidate the mechanism of action on the cell experiments and in vivo.

## Figures and Tables

**Table 1:** Effects of bacterial neuraminidase inhibition of quercetin derivatives (**1**–**4**).

Compounds	IC_50_ ^a^, (μM)	Inhibition Mode	K_i_ ^b^, (μM)
**1**	14.0 ± 0.5	Noncompetitive	13.8 ± 0.2
**2**	25.8 ± 1.0	Noncompetitive	24.7 ± 0.5
**3**	24.3 ± 0.9	Noncompetitive	22.4 ± 0.9
**4**	84.1 ± 3.3	Noncompetitive	79.5 ± 1.8
Quercetin ^c^	26.1 ± 0.6	NT ^d^	NT

^a^ IC_50_ means inhibition rate at 50% of quercetin derivatives against BNA. ^b^ *K*_i_ is the inhibition constant. ^c^ Quercetin was used as the positive control. ^d^ NT was not tested.

**Table 2:** Fluorescence quenching effects of quercetin derivatives (**1**–**4**) against BNA.

Compounds	*K*_SV_ (×10^5^ L mol^−1^)	R^2^	*n*	*K*_A_ (×10^6^ L mol^−1^)	R^2^
**1**	0.0252	0.9954	1.2106	0.05105	0.9938
**2**	0.0144	0.9968	1.1063	0.03994	0.9905
**3**	0.0153	0.9973	1.0959	0.03818	0.9933
**4**	0.0036	0.9172	0.7071	0.00021	0.9991

**Table 3:** Characterization of metabolites from *S*. *pubescens* using LC-TOF/MS.

Peaks	Time (min)	Observed Ion (*m*/*z*)	Calculated Ion (*m*/*z*)	Error (ppm)	Formula	Identification
1	6.7	355.1012	355.1029	−4.79	C_16_H_18_O_9_	chlorogenic acid
2	7.9	181.0499	181.0500	−0.55	C_9_H_8_O_4_	caffeic acid
3	12.9	611.1612	611.1612	0	C_27_H_30_O_16_	rutin
4	13.3	465.1036	465.1033	+0.65	C_21_H_20_O_12_	isoquercitrin
5	14.2	517.1345	517.1346	−0.19	C_25_H_24_O_12_	3,5-dicaffeoylquinic acid
6	15.1	517.1346	517.1346	0	C_25_H_24_O_12_	1,5-dicaffeoylquinic acid
7	15.7	679.1665	679.1663	−0.29	C_34_H_30_O_15_	3,4,5-tricaffeoylquinic acid
8	19.6	317.0642	317.0661	−5.99	C_16_H_12_O_7_	3-*O*-methyl quercetin
9	23.0	331.0798	331.0818	−6.04	C_17_H_14_O_7_	3,4′-*O*-dimethyl quercetin
10	25.8	331.0805	331.0818	−3.93	C_17_H_14_O_7_	3,7-*O*-dimethyl quercetin
11	29.6	345.0961	345.0974	−3.77	C_18_H_16_O_7_	3,7,4′-*O*-trimethyl quercetin

## Data Availability

Not applicable.

## Supplementary Materials

The following supporting information can be downloaded at: https://www.mdpi.com/article/10.3390/molecules28145365/s1, Figures S1–S10: NMR spectra of isolated compounds; Figure S11: Enzyme kinetics of compounds against BNA; Figure S12: LC-Q-TOF/MS analysis of *S. pubescens* extract.

## Sample Availability

Samples of the compounds are available from the authors.
