# Supplementary material for: Inhibitory Potential of Quercetin Derivatives Isolated from the Aerial Parts of Siegesbeckia pubescens Makino against Bacterial Neuraminidase
Source: Molecules. 2023 Jul 12;28(14):5365. doi: 10.3390/molecules28145365 (PMC10386613; doi:10.3390/molecules28145365)
Supplement: Supplementary file 1 [file molecules-28-05365-s001.zip › molecules-2466268-supplementary.pdf]

SUPPLEMENTARY MATERIAL

Inhibitory potential of quercetin derivatives isolated from the aerial parts of *Siegesbeckia pubescens* Makino against bacterial neuraminidase

Yun Gon Son [1], Ju Yeon kim [1], Jae Yeon Park [1], Kwang Dong Kim[2], Ki Hun Park[2], Jeong Yoon Kim [1, *]

[1]Department of Pharmaceutical Engineering, IALS, Gyeongsang National University, Jinju, 52725, Republic of Korea

[2]Division of Applied Life Science (BK21 Four), IALS, ABC-RLRC, Gyeongsang National University, Jinju 52828, Republic of Korea

Correspondence to:

*Corresponding author: Prof. Jeong Yoon Kim, Department of Pharmaceutical Engineering,

IALS, Gyeongsang National University, Jinju, 52725, Republic of Korea

E-mail: jykim21@gnu.ac.kr; Tel.: +82-55-772-3392

List of contents

- 1D, 2D-NMR spectrum of compound **1**

- 1D, 2D-NMR spectrum of compound **4**

- 1H-NMR spectrum of compound **2**

- 1H-NMR spectrum of compound **3**

- Enzyme kinetics of compounds **2-4**

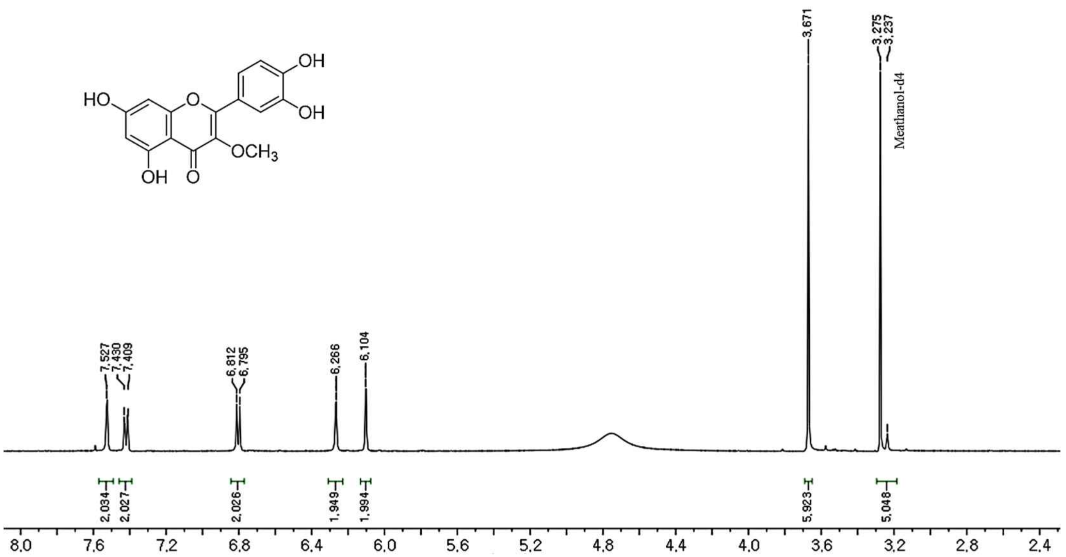

Figure S1. ^{1}H-NMR spectrum of compound **1** (500 MHz, Methanol-d_4)

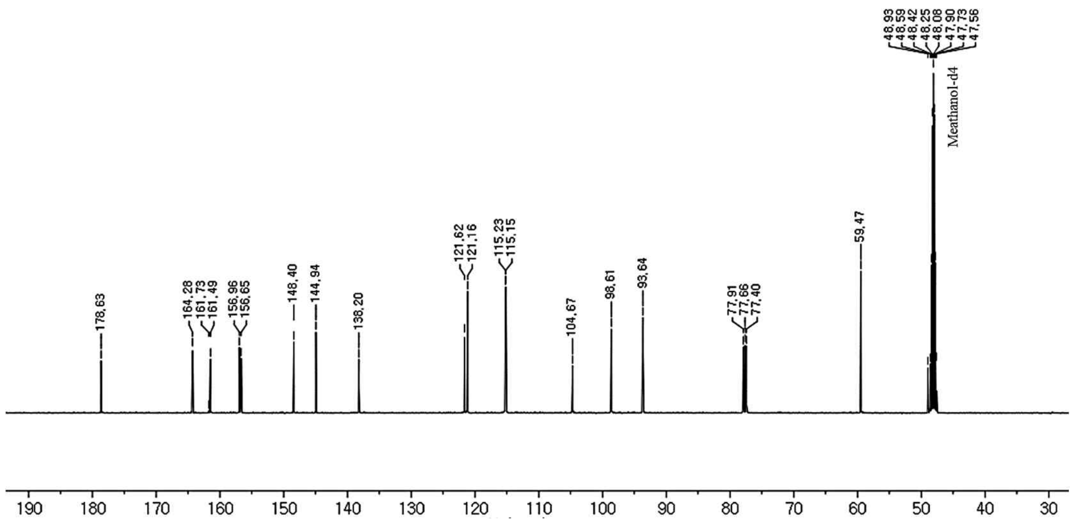

Figure S2. ^{13}C-NMR spectrum of compound **1** (125 MHz, Methanol-d_4)

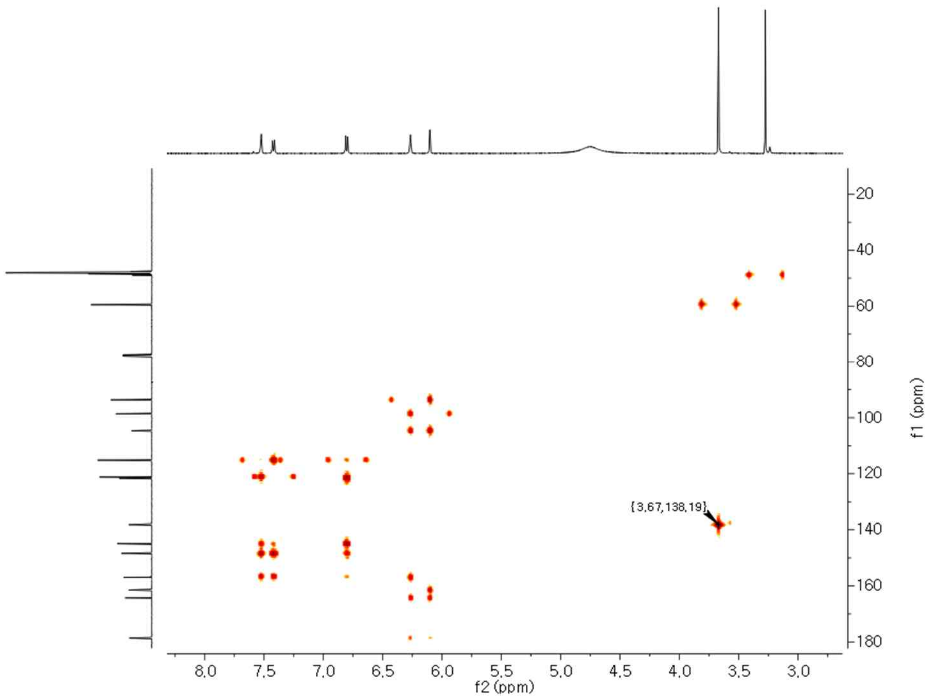

Figure S3. HMBC spectrum of compound **1** (Methanol-d_4)

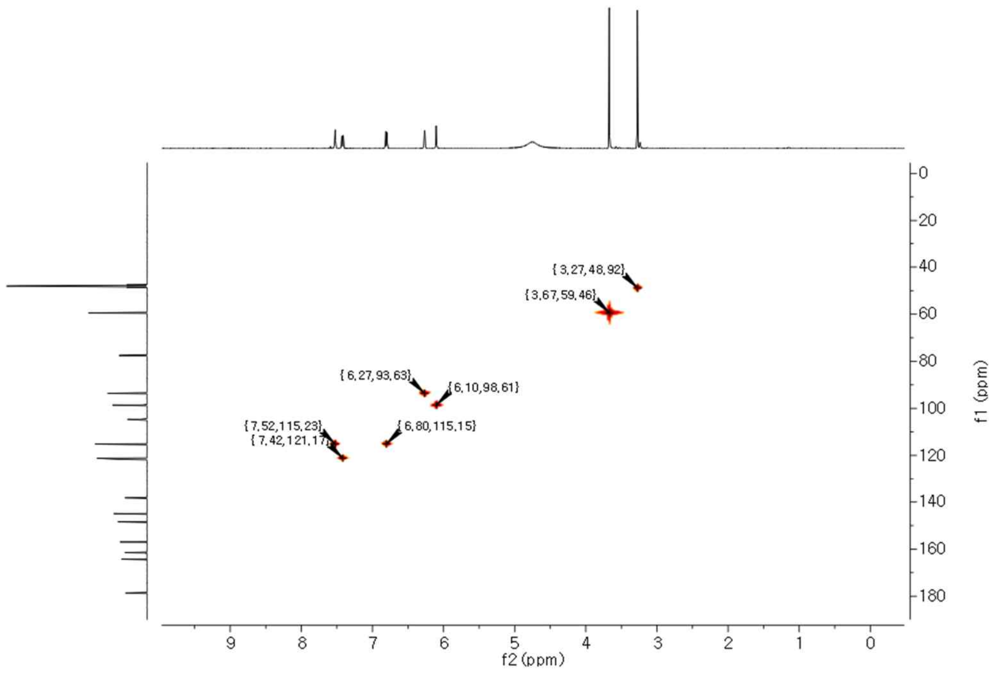

Figure S4. HMQC spectrum of compound **1** (Methanol-d_4)

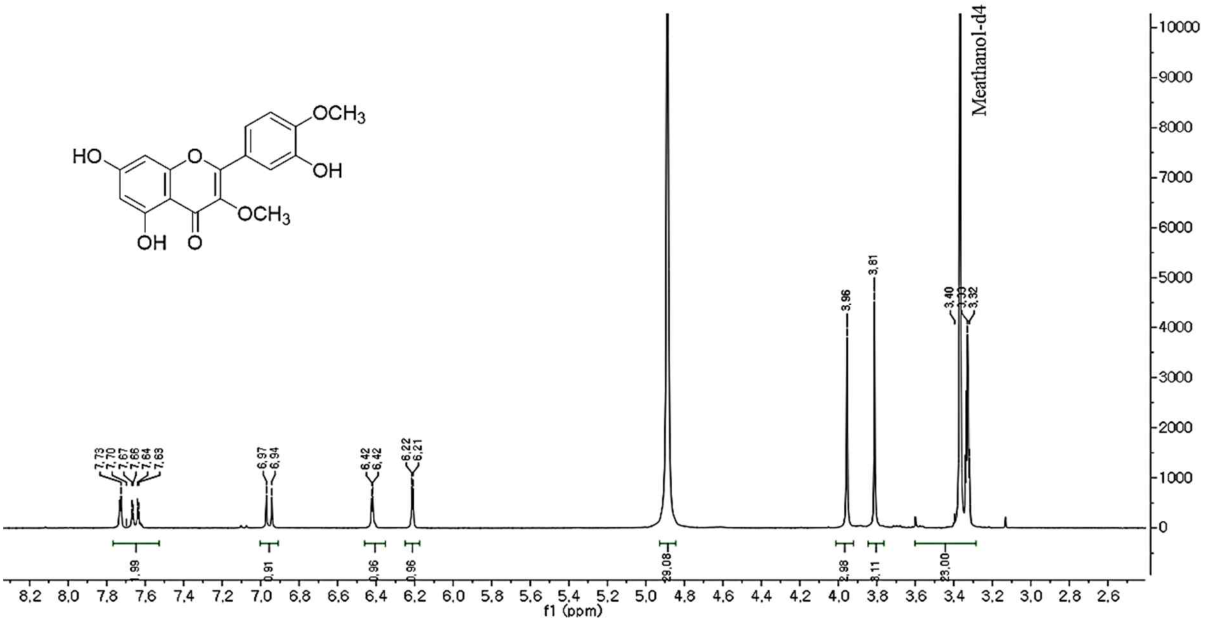

Figure S5. ^{1}H-NMR spectrum of compound **2** (300 MHz, Methanol-d_4)

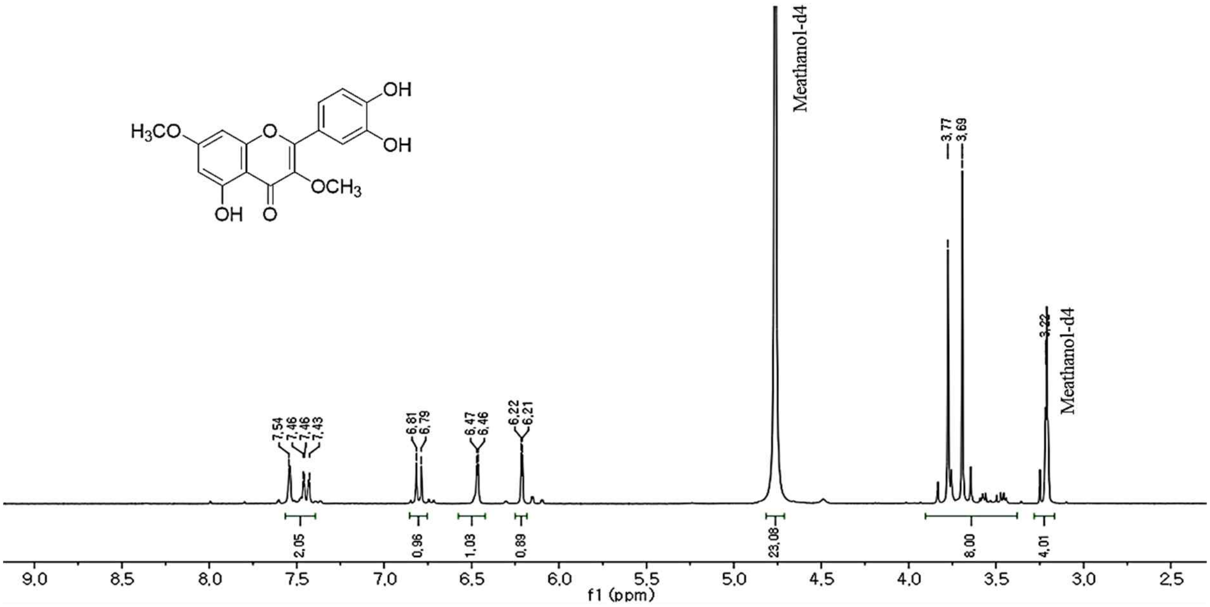

Figure S6. ^{1}H-NMR spectrum of compound **3** (300 MHz, Methanol-d_4)

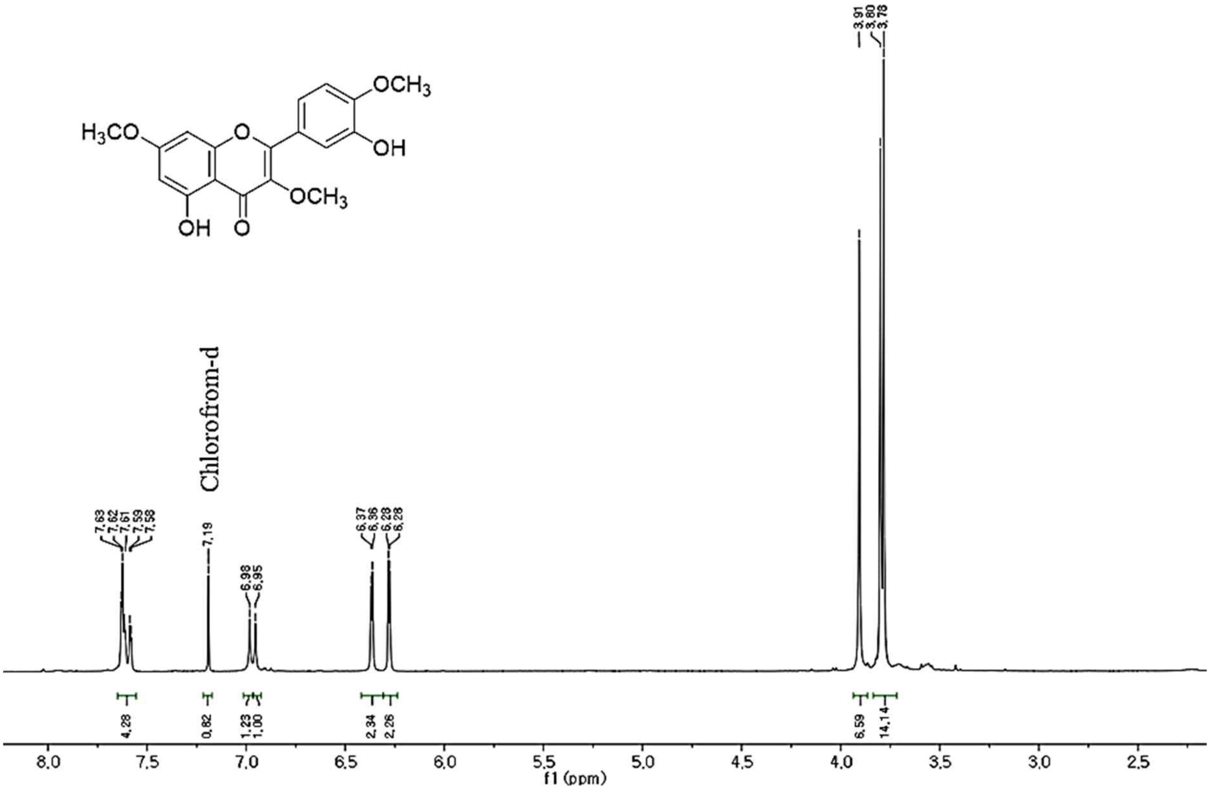

Figure S7. ^{1}H-NMR spectrum of compound **4** (300 MHz, Chloroform-*d*)

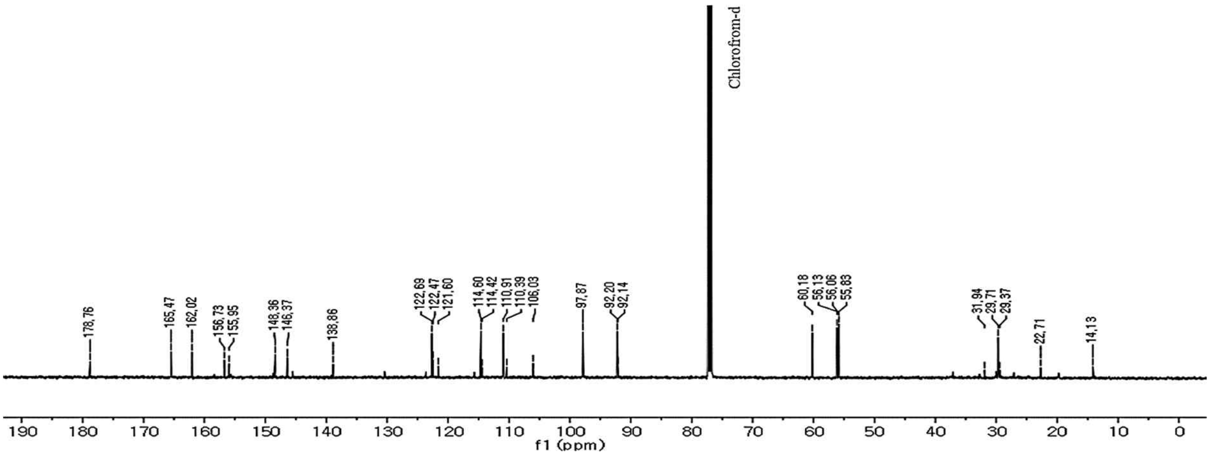

Figure S8. ^{13}C-NMR spectrum of compound **4** (125 MHz, Chloroform-*d*)

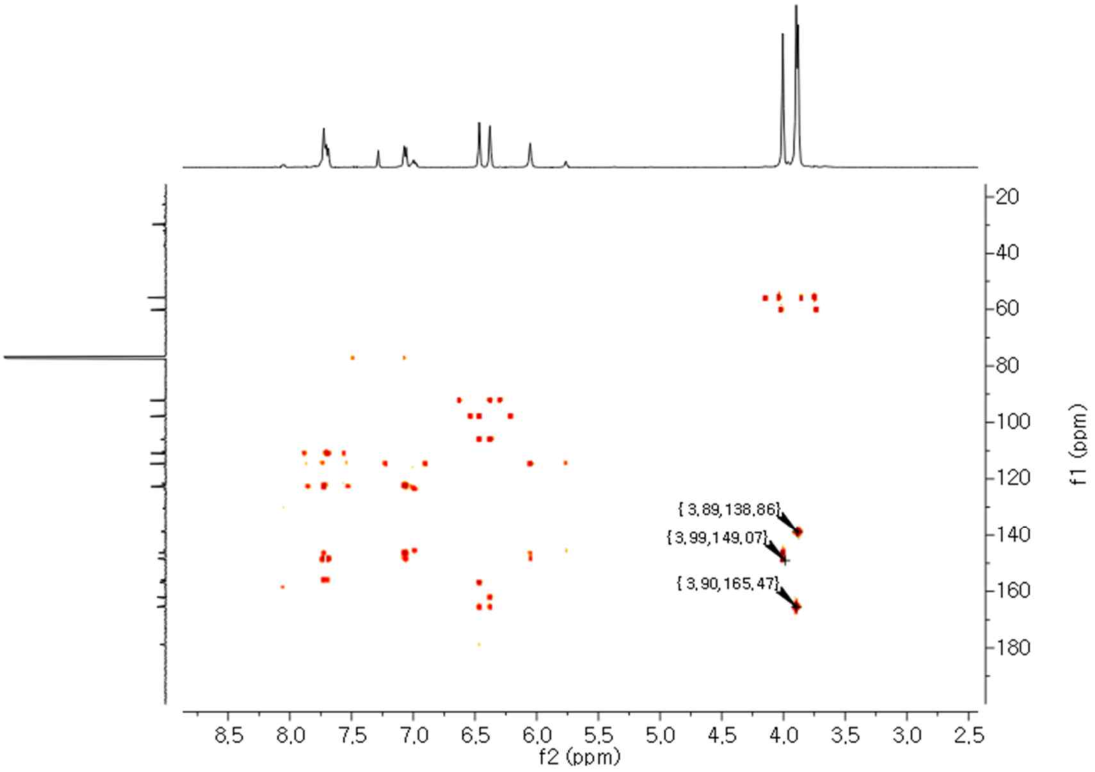

Figure S9. HMBC spectrum of compound **4** (Chloroform-*d*)

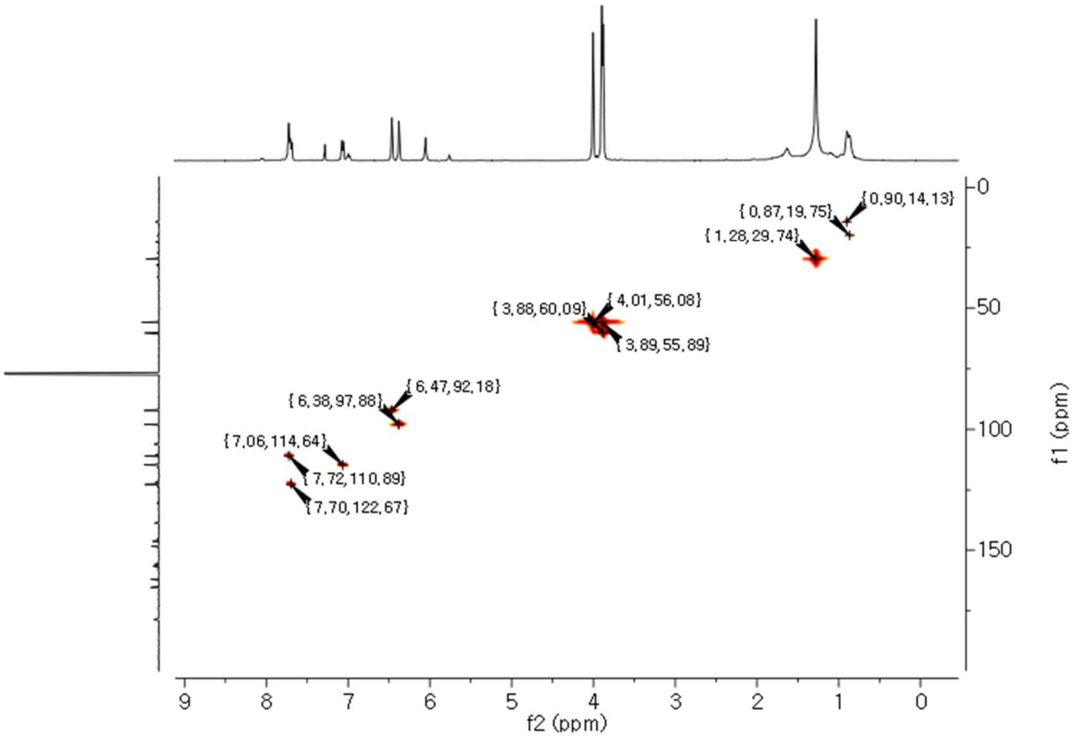

Figure S10. HMQC spectrum of compound **4** (Chloroform-*d*)

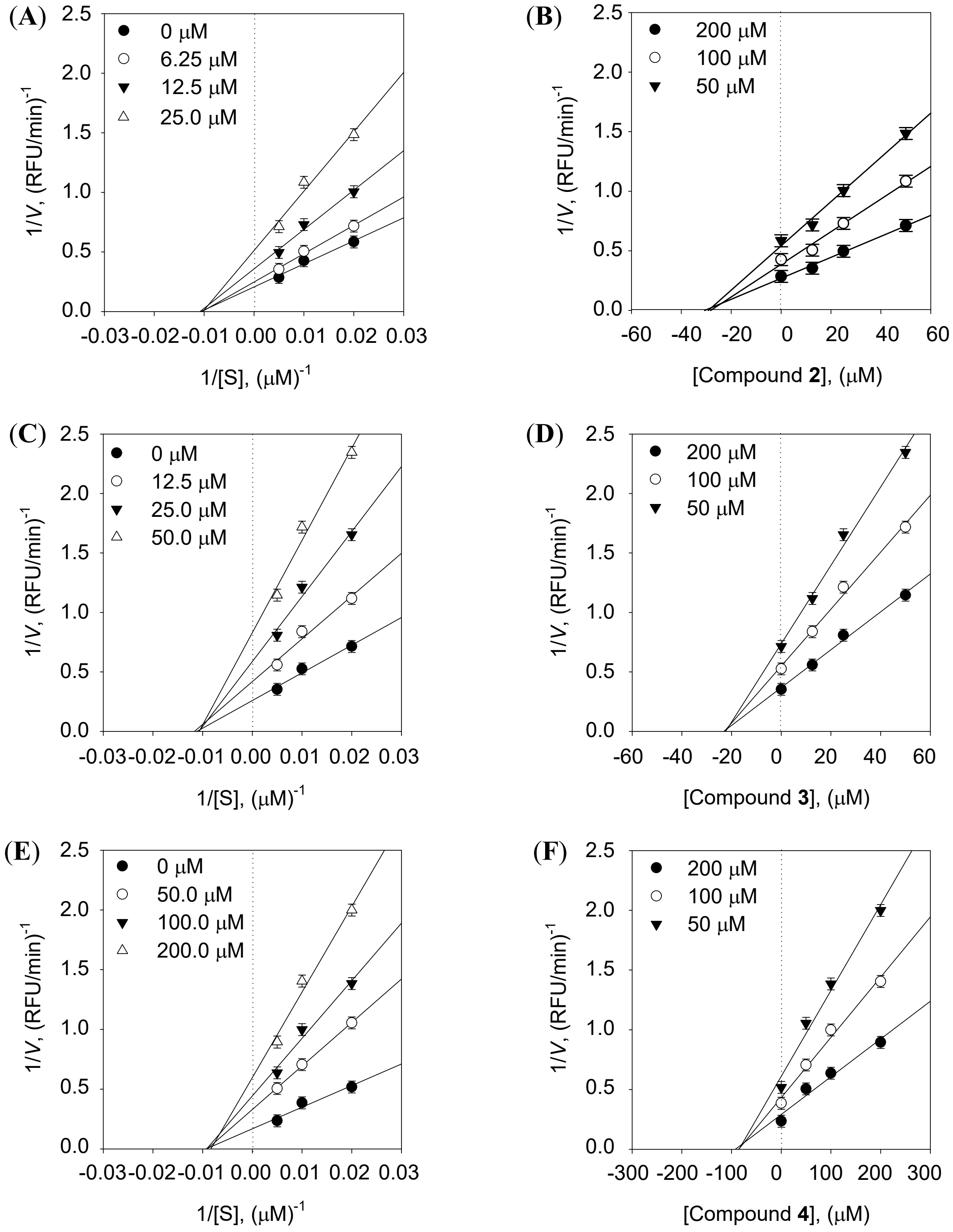

Figure S11. Enzyme kinetics of compounds 2-4 against BNA. Lineweaver-Burk plot of (**A**) compound **2**, (**C**) compound **3**, and (**E**) compound **4**. Dixon plot of (**B**) compound **2, (D)** compound **3**, and (**F**) compound **4**.

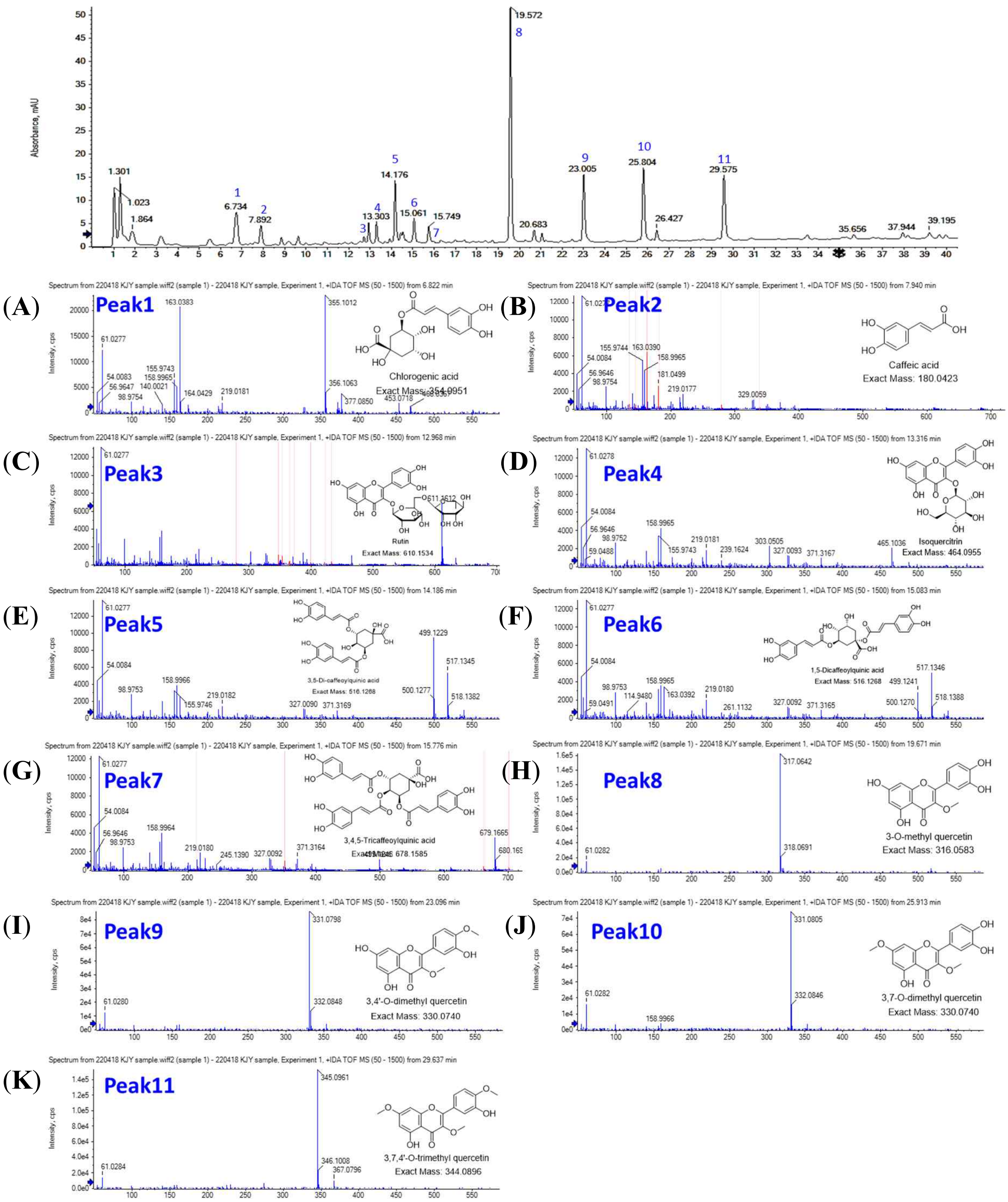

Figure S12. BPI gram of S. pubescens extract and individual mass spectra by LC-Q-TOF/MS analysis (**A**) Chlorogenic acid, (**B**) Caffeic acid, (**C**) Rutin, (**D**) Isoquercitrin, (**E**) 3,5-Dicaffeoylquinic acid, (**F**) 1,5-Dicaffeoylquinic acid, (**G**) 3,4,5-Tricaffeoylquinic acid, (**H**) 3-O-methyl quercetin, (**I**) 3,4′-O-dimethyl quercetin, (**J**) 3,7-O-dimethyl quercetin, and (**K**) 3,7,4′-O-trimethyl quercetin.
